# Long-term trend of and correlation between vegetation greenness and climate variables in Asia based on satellite data

**DOI:** 10.1016/j.mex.2018.07.006

**Published:** 2018-07-18

**Authors:** Munkhnasan Lamchin, Woo-Kyun Lee, Seong Woo Jeon, Sonam Wangyel Wang, Chul Hee Lim, Cholho Song, Minjun Sung

**Affiliations:** Division of Environmental Science and Ecological Engineering, Korea University, Seoul, 02855, South Korea

**Keywords:** Temporal trend analysis, Statistical analysis, Vegetation greenness, Precipitation, Evaporation, Temperature, Correlation, Trend

## Abstract

Satellite data has been used to ascertain trends and correlations between climate change and vegetation greenness in Asia. Our study utilized 33-year (1982–2014) AVHRR-GIMMS (Advanced Very High Resolution Radiometer–Global Inventory Modelling and Mapping Studies) NDVI3g and CRU TS (Climatic Research Unit Time Series) climate variable (temperature, rainfall, and potential evapotranspiration) time series. First, we estimated the overall trends for vegetation greenness and climate variables and analyzed trends during summer (April–October), winter (November–March), and the entire year. Second, we carried out correlation and regression analyses to detect correlations between vegetation greenness and climate variables. Our study revealed an increasing trend (0.05–0.28) in temperature in northeastern India (bordering Bhutan), Southeast Bhutan, Yunnan Province of China, Northern Myanmar, Central Cambodia, northern Laos, southern Vietnam, eastern Iran, southern Afghanistan, and southern Pakistan. However, a decreasing trend in temperature (0.00 to −0.04) was noted for specific areas in southern Asia including Central Myanmar and northwestern Thailand and the Guangxi, Southern Gansu, and Shandong provinces of China. The results also indicated an increasing trend for evapotranspiration and air temperature accompanied by a decreasing trend for vegetation greenness and rainfall. Increases in both the mean annual signal and annual cycle occurred in the forest, herbaceous, and cropland areas of India, Northwest China, and eastern Kazakhstan. The temperature was found to be the main driver of the changing vegetation greenness in Kazakhstan, northern Mongolia, Northeast and Central China, North Korea, South Korea, and northern Japan, showing an indirect relationship (R = 0.84–0.96).

• Temperature is the main climatic variable affecting vegetation greenness.
• A downward trend in vegetation greenness was observed during summer (April–October).
• Temperature showed an upward trend across many areas of Asia during the study period.
• In winter, rainfall showed downward and upward trends in different parts of Asia.

## Graphical abstract

**Figure:**
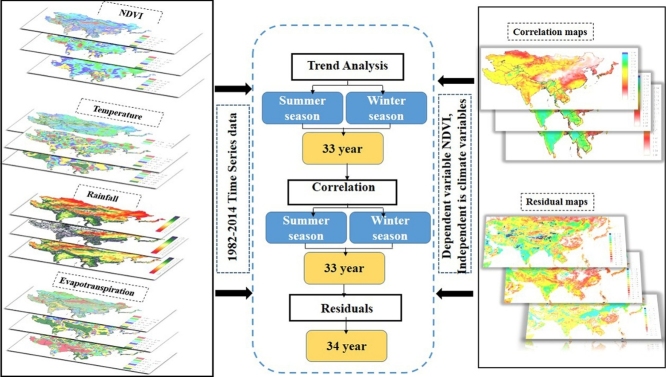

Specifications Table

**Table:** 

Subject area • *Earth and Planetary Sciences* • *Environmental Science*	*Select one of the following subject areas:* • *Agricultural and Biological Sciences* • *Biochemistry, Genetics and Molecular Biology* • *Chemical Engineering* • *Chemistry* • *Computer Science* • *Earth and Planetary Sciences* • *Energy* • *Engineering* • *Environmental Science* • *Immunology and Microbiology* • *Materials Science* • *Mathematics* • *Medicine and Dentistry* • *Neuroscience* • *Pharmacology, Toxicology and Pharmaceutical Science* • *Physics and Astronomy* • *Psychology* • *Social Sciences* • *Veterinary Science and Veterinary Medicine*
More specific subject area	• *Earth and Planetary Sciences* • *Environmental Science*
Method name	*Temporal trend analysis* *Statistical analysis*
Name and reference of original method	*Earth Trends modeler*
Resource availability	

## Method details

### Data

#### Remote sensing vegetation data

The NDVI dataset is the newest version of the Global Inventory Modeling and Mapping Studies (GIMMS) NDVI3g. It is derived from the US National Oceanic and Atmospheric Administration’s (NOAA) Advanced Very High Resolution Radiometer (AVHRR) satellite record. It is the long-term global monthly time series of greenness index, being more than twice as long as those from newer sensors, such as the US National Aeronautics and Space Administration’s (NASA) Moderate Resolution Imaging Spectroradiometer (MODIS, February 2000 until present). The NDVI3g data were collected over a 33-year period, ranging from 1982 to 2014, with a spatial resolution of 8 km, calculated using the average monthly NDVI from January to December. The northern hemisphere latitudes (30° N–90° N) were analyzed in this study.

#### Climate observation data

Monthly average daily maximum temperature (°C), rainfall (mm per month), and potential evapotranspiration (mm per day) data were provided by the Climatic Research Unit Time Series (CRU TS). The current version of CRU TS3.2 was released in 2012. The dataset has a resolution of 0.5° × 0.5°, based on the analysis of over 4000 individual weather station records. A major portion of the input records have been homogenized. However, the dataset itself is not strictly homogeneous. We used the monthly climate data for the same period as the NDVI time series (1982–2014), including the average monthly temperature, rainfall, and evapotranspiration for all the seasons from January to December, totaling 1536 data points from 384 months. We changed the cell size to 0.083, which is the same as NDVI3g data (8 km), with 1319 columns and 598 rows covering all the climate data using Arc GIS.

### Method

#### Temporal trend analysis

The monotonic trend (Mann–Kendall) option provides a nonlinear trend indicator that measures the degree to which a trend is consistently increasing or decreasing. It ranges from −1 to +1. A value of +1 demonstrates a trend that continuously increases and never decreases. The contrary is true when the value is −1 and 0 value is demonstrated no consistent trend.

The trend of the time series data can be computed using a nonparametric technique that was introduced by Theil and developed by Sen. The Theil–Sen (TS) slope estimator is the median of the slopes computed for values observed at all pairwise time steps for a total of *n(n*−*1)/2* slopes. This method has many advantages. The TS technique is robust against outliers and has the ability to reject anomalies without affecting the slope. The number of anomalies that this approach can reject without being affected (known as the breakdown bound) equals approximately 29% of the sample size.

Temporal trends of the datasets were checked by applying linear regression and were used to model the series of monthly NDVI composites as a dependent variable versus the independent variables of potential evapotranspiration, rainfall, and temperature. The analyses were broken into periods covering 1982–2014, as determined by the temporal coverage of the climate and NDVI datasets. The outputs of the trend analyses include maps of relationship coefficients (*R*-values) and slopes, indicating the strengths and magnitudes of the computed trends.

#### Statistical analysis

The relationship coefficient, R, is a measure of the strength of a linear relationship between two variables. The coefficient of definition, *R*^2^, is the proportion of the variation of a response variable that is explained by a fitted statistical model; R^2^ is mostly demonstrated as a percentage. The relationship is computed from the strength of the linear correlation between NDVI and temperature, rainfall, and potential evapotranspiration by computing the per-pixel relationship coefficient (*R*^2^) from 33-years of overlay monthly observations (January 1982–December 2014). The linear association between the NDVI and climatic variables and vegetation growth was determined using single correlation (*R*) and regression (*R*^2^) analyses. An annual time step (starting in January 1982) was used to examine the effects on the climatic driver and vegetation growth reported that local differences in the NDVI and rainfall relationship due to dominating soil and vegetation types and their individual rain use efficiencies were considered by calculating respective intercepts and slopes for each pixel. During the regression analysis, a causal correlation between the dependent NDVI values and independent temperature, rainfall, and potential evapotranspiration variables is calculated; the predicted high NDVI values for each month and each pixel are calculated based on the observed rainfall, temperature, rainfall, and potential evapotranspiration.

### Data analysis

#### Temporal trend analysis

The monotonic trend (Mann-Kendall) option provides a nonlinear trend indicator that measures the degree to which a trend is consistently moving upwards or downwards. It ranges from −1 to +1. A value of +1 indicates a trend that is continuously moving upward, with no downward movement. The contrary is true when the value is −1, while 0 indicates no consistent trend.

**Fig. 1:**
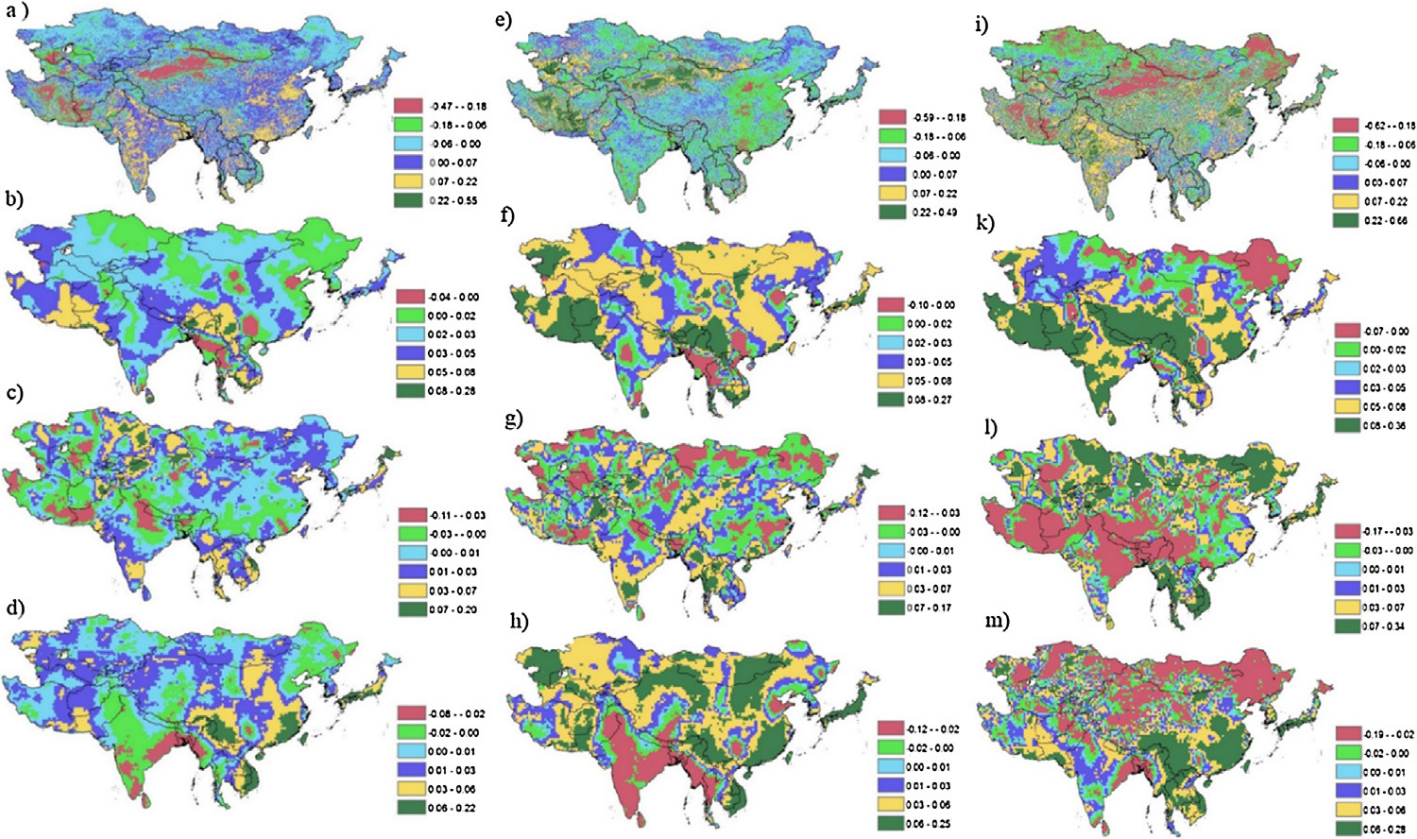
NDVI (a, e, i), temperature (b, f, k), rainfall (c, g, l), and potential evapotranspiration (d, h, m) trends based on Mann-Kendall tests for November–March (left), April–October (middle), and annually (right) during 1982–2014.

The trend of the time series data can be computed using a nonparametric technique that was introduced by and developed by. The Theil-Sen (TS) slope estimator is the median of the slopes computed for values observed at all pairwise time steps for a total of *n (n* − *1)/2* slopes. This method has many advantages. The TS technique is robust against outliers, with the ability to reject anomalies without affecting the slope. The number of anomalies that this approach can reject without being affected (known as the breakdown bound) equals approximately 29% of the sample size (Fig. 1).

#### Statistical analysis

**Fig. 2:**
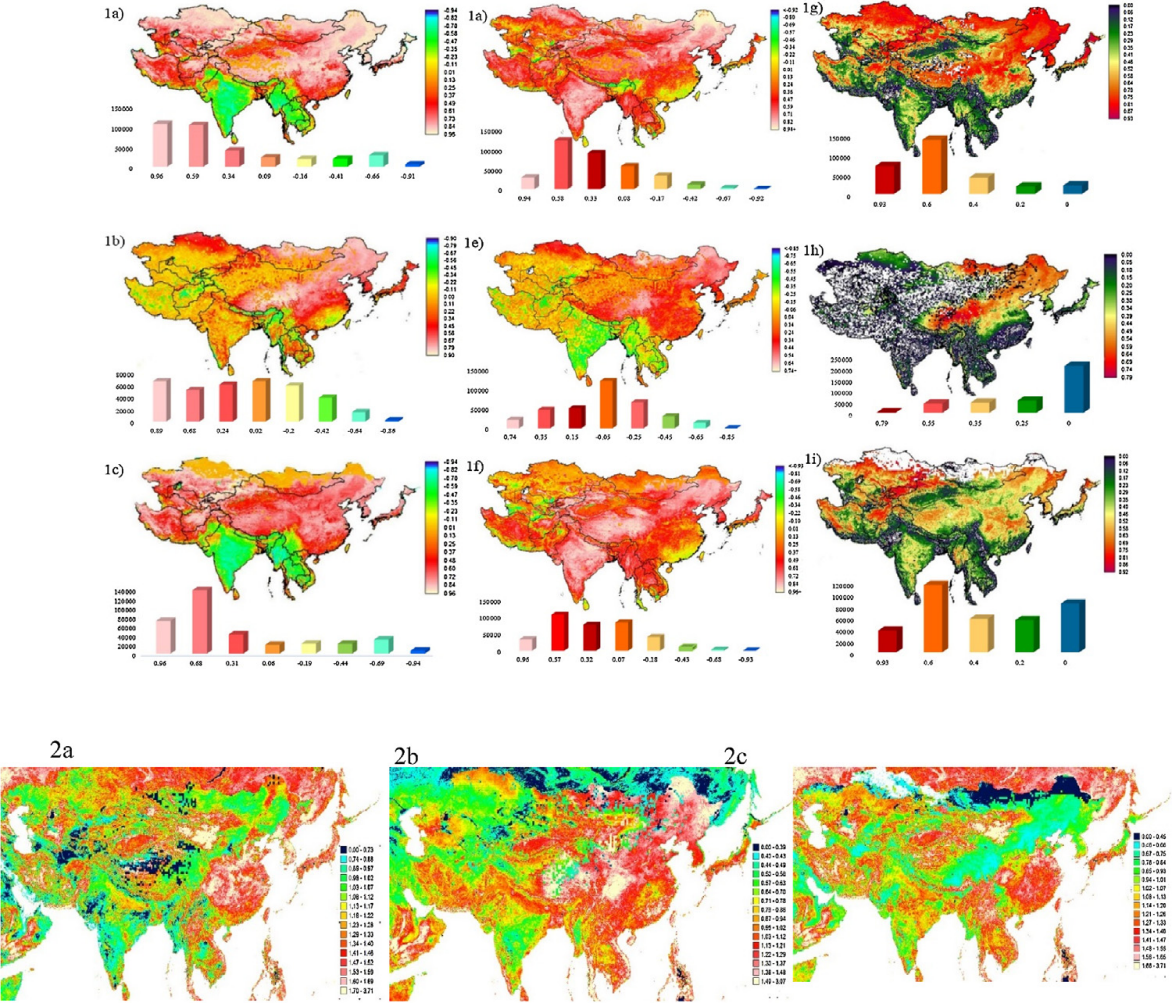
Frequency graph distribution and map of coefficient of determination (R^2^) for all seasons (left); correlations (R) for the growing season from April to October (middle) and for all months (right) for NDVI and temperature (1a, 1d, and 1g), NDVI and rainfall (1b, 1e, and 1h), and NDVI and potential evapotranspiration (1c, 1f, and 1i). Residual maps with the dependent variable of NDVI and the independent variables of temperature (2a), rainfall (2b), and potential evapotranspiration (2c) for 1982–2014.

The linear association of the NDVI and climatic variables with vegetation growth was determined using single correlation (*R*) and coefficient of determination (*R*^2^) of regression analyses. The relationship coefficient, R, is a measure of the strength of a linear relationship between two variables. The coefficient of determination, *R*^2^, is the proportion of the variation of a response variable that is explained by a fitted statistical model. R^2^ is mostly demonstrated as a percentage. The relationship is computed based on the strength of the linear correlation of NDVI with temperature, rainfall, and potential evapotranspiration, by computing the per-pixel relationship coefficient (*R*^2^) from 33 years of overlay monthly observations (January 1982–December 2014). For identifying the local differences more clearly, residual maps with the dependent variable of NDVI and independent variables of temperature, rainfall, and potential evapotranspiration were created (Fig. 2).

